# Mitochondria Targeted Antioxidant Significantly Alleviates Preeclampsia Caused by 11β-HSD2 Dysfunction via OPA1 and MtDNA Maintenance

**DOI:** 10.3390/antiox11081505

**Published:** 2022-07-31

**Authors:** Jing Long, Yan Huang, Zhengshan Tang, Yali Shan, Dou Feng, Wenqin Wang, Juan Liu, Ying Huang, Hang Gu, Dewei Guo, Ruojin Yao, Xin Ni

**Affiliations:** 1 Department of Gynecology and Obstetrics, Xiangya Hospital Central South University, Changsha 410008, China; longjingrenee@163.com (J.L.); s1970453015@163.com (Y.S.); jane701180@163.com (D.F.); guodewei@csu.edu.cn (D.G.); ruojinyao@aliyun.com (R.Y.); 2 National International Joint Research Center for Medical Metabolomics, Xiangya Hospital Central South University, Changsha 410008, China; tangzhengshan1992@163.com (Z.T.); wangwenqin_xy@163.com (W.W.); liujuancsusky@163.com (J.L.); 3 Department of Physiology, Navy Medical University, Shanghai 200433, China; huangyantiger2005@163.com; 4 Maternity and Child Health Hospital of Pudong New District, Shanghai 201206, China; stassi22@163.com; 5 Department of Gynecology and Obstetrics, Changhai Hospital, Shanghai 200433, China; guhh@sina.com

**Keywords:** preeclampsia, mitochondria, placenta, OXPHOS, mtDNA

## Abstract

We have previously demonstrated that placental 11β-hydroxysteroid dehydrogenase type 2 (11β-HSD2) dysfunction contributes to PE pathogenesis. We sought to elucidate molecular mechanisms underlying 11β-HSD2 dysfunction-induced PE and to seek potential therapeutic targets using a 11β-HSD2 dysfunction-induced PE-like rat model as well as cultured extravillous trophoblasts (EVTs) since PE begins with impaired function of EVTs. In 11β-HSD2 dysfunction-induced PE-like rat model, we revealed that placental mitochondrial dysfunction occurred, which was associated with mitDNA instability and impaired mitochondrial dynamics, such as decreased optic atrophy 1 (OPA1) expression. MitoTEMPO treatment significantly alleviated the hallmark of PE-like features and improved mitDNA stability and mitochondrial dynamics in the placentas of rat PE-like model. In cultured human EVTs, we found that 11β-HSD2 dysfunction led to mitochondrial dysfunction and disrupted mtDNA stability. MitoTEMPO treatment improved impaired invasion and migration induced by 11β-HSD2 dysfunction in cultured EVTs. Further, we revealed that OPA1 was one of the key factors that mediated 11β-HSD2 dysfunction-induced excess ROS production, mitochondrial dysfunction and mtDNA reduction. Our data indicates that 11β-HSD2 dysfunction causes mitochondrial dysfunctions, which impairs trophoblast function and subsequently results in PE development. Our study immediately highlights that excess ROS is a potential therapeutic target for PE.

## 1. Introduction

Preeclampsia (PE) impacts 5% to 8% of pregnancies worldwide and causes maternal morbidity and mortality [1]. It is now generally accepted that PE begins with impaired functions of extravillous trophoblasts (EVTs) and abnormal placentation with subsequent release of antiangiogenic markers, mediated primarily by soluble fms-like tyrosine kinase-1 (sFlt-1) and soluble endoglin (sEng). High levels of sFlt-1 and sEng lead to endothelial dysfunction, vasoconstriction, and immune dysregulation, which can negatively impact every maternal system and the fetus [2]. Underlying mechanisms contributing to the pathophysiology of PE are poorly understood so far. Given that placenta is the principal contributor to PE development, identification of the key pathways related to PE in placenta is an important approach for seeking new intervention strategy. 

11β-hydroxysteroid dehydrogenase type 2 (11β-HSD2) converts cortisol (corticosterone in rodents), the active glucocorticoids (GCs) form, into inert 11-keto derivatives (cortisone, 11-dehydrocorticosterone), thereby controlling the concentration of active glucocorticoids. In placenta, 11β-HSD2 determines local and distant events, i.e., controls GCs availability locally and the amount of maternal GCs across the placenta [3]. GCs are involved in many events during pregnancy including embryo implantation, growth and development of the fetus and placenta as well as initiation of parturition [4,5]. We have recently demonstrated that placental 11β-HSD2 dysfunction is one of key mechanisms underlying PE pathogenesis by showing that 11β-HSD2 dysfunction leads to excess GCs accumulation and subsequently results in an impaired invasion and migration function of EVTs in human cell models. Moreover, inhibition of placental 11β-HSD2 by carbenoxolone (CBX) leads to abnormal spiral artery remodeling and placentation and subsequently results in PE features in the pregnant rat model [6]. Thus, the rat PE-like model of placental 11β-HSD2 dysregulation is close to human PE characteristics and can be an ideal model for investigation of the pathogenesis and the potential therapeutic strategies. We have previously demonstrated that placental 11β-HSD2 dysfunction promotes sFlt-1 release via up-regulation of a disintegrin and metalloprotease (ADAM)17 expression [6]. However, the molecular mechanisms underlying PE pathogenesis associated with placental 11β-HSD2 dysfunction remain largely unknown.

The purposes of the present study were to elucidate the molecular mechanisms underlying 11β-HSD2 dysfunction-mediated PE features and seek valuable therapeutic targets. To achieve these goals, we conducted a series of experiments. We first comprehensively assessed the molecular network in placentas of the rat PE-like model by using RNA-seq analysis combined with metabolomics and found that mitochondrial dysfunction was a key event in placentas of this model. Given that excess reactive oxygen species (ROS) is a key event in mitochondrial dysfunction, we hypothesized that excess mitochondrial ROS production plays an important role in PE pathogenesis associated with 11β-HSD2 dysfunction. We subsequently investigated the effects of mitochondria-targeted antioxidant mitoTEMPO on PE-like features in the rat model and explored potential molecular mechanisms. Given that impaired EVT functions lead to abnormal spiral artery remodeling and placentation, the roles of mitochondria in EVT functions require study. Although there are some differences in pregnant maintenance between rodents and humans, the processes of placental development and spiral artery remodeling are similar in many aspects [7]. We therefore investigated whether mitochondrial dysfunction plays key roles in impaired EVT functions caused by 11β-HSD2 dysfunction in the model of cultured human EVTs. Finally, we sought to clarify the key molecular mechanism responsible for 11β-HSD2 dysfunction-mediated abnormal trophoblast function.

## 2. Materials and Methods

### 2.1. Animals

All experimental steps were supported by the Ethical Committee of Medical Research of Xiangya Hospital Central South University (Changsha, China).

Adult Sprague–Dawley rats (250–300 g) were obtained from Hunan SLAC Laboratory Animal Co., (Changsha, China). The animals were housed in a standardized environment (25 °C, 55% humidity, 12 h daylight and 12 h day/night light cycles). Female and male rats were mated overnight, and the following day was counted as gestational day (GD) 0.5. From GD 7.5 to GD 17.5, pregnant rats were administered (s.c) with CBX at 2.4 mg/kg (*n* = 8), mitoTEMPO (i.p) at 1 mg/kg (*n* = 8) and CBX combined with mitoTEMPO (*n* = 8) once a day, respectively. Carbenoxolone disodium salt (98% purity) and mitoTEMPO were purchased from Sigma-Aldrich and dissolved in saline. Control rats (*n* = 8) were injected with same volume of saline. The dosage of CBX was chosen based on our previous study [6], while the dosage of mitoTEMPO was chosen based on the study by Vaka et al. [8] and our preliminary study. All the rats received measurement of blood pressure as described previously [6]. Doppler ultrasonography was conducted on GD 19.5. On GD 20.5, after measuring blood pressure, the animals were sacrificed by deep anesthetization, and maternal blood, pups, placentas, and kidney tissues were collected. Several blood samples were discarded due to hemolysis, and all the qualified serum samples were used for determination of fms-like tyrosine kinase 1 (sFlt1) and soluble endoglin (sEng) levels. Among placental samples, two placentas from two dams in each group were used for electron microcopy. Eight placentas were picked up in each group (at least 1 placenta from each dam) for untargeted metabolomics analysis, and 6 placentas were obtained from 6 dams in each group for targeted metabolomic analysis; 5 samples were obtained from 5 dams in each group for RNA-seq analysis. Other placentas were used for morphology analysis, determination of mitochondrial function and mRNA and protein levels of targeted genes. 

Nonpregnant female rats were randomly divided into two groups, receiving saline or CBX (*n* = 5 in each group). The rats were injected with saline or CBX at 2.4 mg/kg once a day for 10 days. After measurement of arterial blood pressure, kidney tissues were collected. 

### 2.2. Measurement of Blood Pressure

Mean arterial pressure (MAP) was monitored by the noninvasive tail-cuff system (BP-300A automatic noninvasive blood pressure measurement system, Techmen) in each dam from GD 9.5 to 17.5 every two days as described previously [6]. 

Arterial blood pressure was recorded through the right carotid artery of pregnant rats on GD 20.5 and nonpregnant rats as described previously [6]. Briefly, the pregnant rats were anaesthetized (urethane 800 mg/kg and alpha-chloralose 40 mg/kg, i.p.), and the right carotid artery was catheterized. Then, systolic blood pressure (SBP) and MAP was recorded by the computer collecting and disposing system for organic signals (BL-420N, Techmen software Ltd., Chengdu, China). Among pregnant rats, successful measurements were obtained in 5 rats with CBX treatment, 6 rats with CBX + mitoTEMPO treatment, and 8 rats with vehicle treatment. Successful determination was obtained in all nonpregnant rats. All successful BP measurements were used for the statistical analysis. 

### 2.3. Measurement of Uteroplacental Blood Flow

Uteroplacental blood flow was determined by Doppler ultrasonography in pregnant rats on GD 19.5 as described previously [6,9]. Briefly, the rats were anaesthetized with 3% isoflurane in air by face mask. After removal of abdominal hairs, a high frame rate 22 mHz probe (Vevo 2100, FuJiFilm) was used to detect waveform. In each dam, measurements were performed on 2–4 implantation sites. In each implantation site, the Doppler waveforms of 2–3 spiral arteries (SAs), maternal canal and umbilical artery (UmbA) were recorded, and the peak systolic velocity (PSV) was measured. Finally, mean PSV of umbilical artery and maternal canal and spiral arteries from two implantation sites were obtained in each pregnant rat. 

### 2.4. Analysis of Protein and Creatinine Concentration in Urine

The protein concentration in urine was determined as described by prior studies [6,10]. Briefly, single-void urine specimens were gained individually in metabolic cage in each dam on GD 18.5–19.5 under fasting conditions. Total protein concentration was measured by BCA protein assay kit (ECOTOP Biotechnology, Guangzhou, China). Creatinine levels were measured by creatinine assay kit (Beyotime Technology, Shanghai, China). 

### 2.5. Histological Morphology of Kidney 

Renal morphology examination was performed in each pregnant rat. Kidney paraffin sections (3 µm) were de-waxed and hydrated and stained with Hematoxylin and Eosin (H&E) and periodic acid-Schiff (PAS) for glomerular pathological evaluation as described previously [6,11] and after consultation with an experienced renal pathologist at Xiangya Hospital.

### 2.6. Histological Morphology and Immunostaining of Placenta

Morphology analysis was conducted on a placenta in each pregnant rat as described previously [6]. Briefly, placenta paraffin sections (5 µm) were de-waxed and hydrated for H&E stain and immunostaining. Trophoblasts and smooth muscle cells in placentas and uterus were identified by immunocytochemistry as described previously [4]. The sections were incubated with antibodies against cytokeratin 7 (CK7) (1:500; DAKO) or α smooth muscle actin (α-SMA) (1:400; Servicebio) overnight at 4 °C. Negative controls consisted of the sections in which the primary antibody was substituted with an equal concentration of mouse IgG (DAKO). The bound antibodies were detected with DAB Horseradish Peroxidase Color Development Kit (ZCIBIO Technology, Shanghai, China). Counterstaining was performed with hematoxylin. 

### 2.7. Electron Microscopy

The fresh rat placental tissues were immersed in a 4% solution of paraformaldehyde in PBS and were fixed in this solution for 24 h. Then, tissues were post fixed with osmium tetraoxide, dehydrated in a graded ethanol series, and embedded in epoxy resin. Samples were sectioned (50 nm), and subsequently counterstained with uranyl acetate and lead citrate and observed under a Hitachi H-7700 Transmission Electron Microscope (Hitachi, Tokyo, Japan). Morphology of mitochondria in the labyrinth zone was examined by a blinded observer. 

### 2.8. Transcriptome Sequencing Analysis

RNA-sequencing was performed by Novogene Co., Ltd. (Beijing, China). Briefly, FPKM of each gene was calculated based on the length of the gene and read counts mapped to this gene. Subsequently, differential expression analysis of two groups was performed using the DESeq2 R package (1.20.0). DESeq2 provides statistical routines for determining differential expression in digital gene expression data using a model based on the negative binomial distribution. The resulting *p*-values were adjusted using Benjamini and Hochberg’s approach for controlling false discovery rates. Genes with an adjusted *p*-value < 0.05 and fold change > 1.5 were assigned as differentially expressed. Next, Novomagic cloud platform was used for functional enrichment analysis.

### 2.9. Untargeted and Targeted Metabolomics 

Untargeted metabolomics were performed on an Ultimate 3000 UHPLC (Thermo Fisher, Waltham, MA, USA) system coupled to a Q Exactive HF (Thermo Fisher, Waltham, MA, USA). Before LC-MS/MS analysis, samples were prepared for extraction. Briefly, 30 mg samples were cut into pieces and added to 120 L pre-chilled mixed solution (MeOH: ACN: H_2_O = 5:3:2). The mixtures were homogenized for 5 min and shaken for 1.5 h at 4 °C. Then the supernatants were obtained by centrifugation at 16,000× *g* for 10 min at 4 °C. A two microliter (2 mLaliquot of sample was injected into a Waters CORTECS T3 column (1.6 m, 3 mm,150 mm) operating at 35 °C. The mobile phase A was 0.1% formic acid and B was methanol for POS mode; mobile phase C was 5 mM NH_4_-HCO_3_ and B was methanol for NEG mode. MzCloud (Thermo Fisher Scientific) was used to obtain the exact mass (to 5 decimal places) of endogenous substances and to identify the substances based on the mass and molecular formula. Then, the collected information (exact mass and retention time) of endogenous substances was inserted into TraceFinder (Thermo Fisher Scientific) for qualitative analysis of the peak area of each substance in the sample. Subsequently, Principal Component Analysis (PCA) was performed using Simca-p 14.1, and differential metabolites with VIP > 1, *p* value < 0.05 and Fold Change > 1.5 were identified. Next, MetaboAnalyst 5.0 (https://www.metaboanalyst.ca/ (accessed on 1 May 2021)) was used to analyze the differential metabolites, and subsequently elucidate metabolic pathways. 

Targeted metabolomics of central carbon metabolism was performed by Novogene Co., Ltd. The detection of the experimental samples using Multiple Reaction Monitoring (MRM) was based on Novogene in-house database. The Q3 were used for the metabolite quantification. The Q1, Q3, retention time (RT), declustering potential (DP) and collision energy (CE) were used to the metabolite identification. The data files generated by HPLC-MS/MS were processed using the SCIEX OS Version 1.4 to integrate and correct the peak. The main parameters were set as follows: minimum peak height, 500; signal/noise ratio, 5; gaussian smooth width, 1. The area of each peak represents the relative content of the corresponding substance. 

### 2.10. Mitochondrial Isolation and Function Determination

One placenta from each pregnant rat was used for mitochondrial function analysis. Mitochondria were isolated from fresh placental tissues following the protocol of the Kit (Beyotime). The mitochondria were divided into three parts, one of which was stored at −80 °C that may be needed in the future, and another wass used to determine the protein concentration for subsequent quantitative analysis. One was used for the mitochondrial function assay. ROS, ATP, and mitochondrial membrane potential (MMP) was assessed using the MitoSOX™ Red mitochondrial superoxide indicator (Invitrogen), enhanced ATP Assay Kit (Beyotime), and enhanced MMP assay kit with JC-1 (Beyotime) according to manufacturer’s instruction, respectively.

### 2.11. Enzyme-Linked Immunosorbent Assay (ELISA)

The concentrations of sFlt1 and sEng in the circulation were determined by using a commercial ELISA kit from ZCIBIO Technology Co., Ltd. (Shanghai, China) according to the manufacturer’s instructions.

### 2.12. Total RNA Extraction and Quantitative Real-Time PCR (Q-PCR)

One placenta from each pregnant rat was used for total RNA extraction. Cultured cell samples were collected after incubation. TRIzol reagent (AGBIO Co., Ltd., Changsha, China) was used to extract total RNAs from cells and the frozen tissues. 1 µg RNA was reverse transcribed to generate cDNA by using PrimeScript RT Master Mix Kit (TaKaRa Bio.Inc, Dalian, China). The primers used for Q-PCR were synthesized by Tsingke Biotechnology (Beijing, China). They were listed in Table S1. Q-PCR reaction was carried out on MiniOpticon™ Real-Time PCR Detection System (BioRad, Hercules, CA, USA). The reaction solution consisted of 2.0 μL diluted cDNA, 0.2 μM of each paired primer and 1×ChamQ Universal SYBR qPCR Master Mix (Vazyme Biotechnology, Nanjing, China). The housekeeping gene β-actin was used for as an internal control. The specificity of PCR products was examined by the melting curve at the end of the amplification and subsequent sequencing. To determine the relative quantitation of gene expression for both target and housekeeping genes, the comparative Ct (threshold cycle) method with arithmetic formulae (2 −^ΔΔCt^) was used. 

### 2.13. Mitochondrial DNA Copy Number Detection

Relative levels of mitochondrial DNA (mtDNA copy number) were determined as described by Lagouge et al. [12]. Briefly, total DNA was extracted from cells or from placental tissues using a Universal Genomic DNA Kit (TaKaRa). The mtDNA specific PCR (16S rRNA) was used to measure mtDNA copy number and was normalized to the nuclear ribosomal protein s18 (RPS18) for rat and nuclear nuclear β-2 microglobulin (B2M) for human cells. The primers are listed in Table S1.

### 2.14. Western Blotting Analysis

One placenta picked from each dam was used for determination of protein expression of targeted genes. 30 mg of placental tissues was homogenized in cold RIPA lysis buffer containing protease inhibitor cocktail (ECOTOP). After incubation, the cultured cells were scraped off the plate in the presence of the above lysis buffer. The protein concentration was quantified by BCA kit (ECOTOP). Equivalent amounts of protein were separated by SDS-PAGE, and subsequently transferred to PVDF membranes (Merck–Millipore). After incubation with blocking buffer of 5% skim milk powder, the membranes were incubated with specific primary antibodies overnight at 4 °C. The antibodies used are listed in Table S2. After washing, the membranes were incubated with corresponding secondary antibodies for 1 h. After washing, the enhanced chemiluminescence Western blotting detection system (Vazyme Biotechnology, Nanjing, China) was then used to visualize the reacting bands. The intensities of light-emitting bands were detected and quantified using Tanon 4600SF Image system (Shanghai Tanon. Ltd., Shanghai, China). Semi quantification of the protein bands density was carried out using Image J software. To control sampling errors, the ratio of band intensities to β-actin was obtained to quantify the relative protein expression level.

### 2.15. Cell Culture and RNA Interference

HTR8 is an EVT cell line originally from human first-trimester placenta (a gift from Prof. Charles H. Graham, Queen’s University, Kingston, ON, Canada). The cells were maintained in DMEM containing 10% fetal calf serum (FCS), penicillin (100 U/mL) and streptomycin (100 mg/mL) at 37 °C in 5% CO_2_-95% air humidified atmosphere. Cells were treated with cortisol (10^−6^ M) in the presence or absence of CBX (10^−6^ M) or combined with mitoTEMPO (10^−7^ M) for 24 h to 72 h, and then the cells were harvested for determination of mtDNA content, mRNA and protein levels of the factors controlling mitochondrial dynamics. The dosages of cortisol and CBX were used based on our previous study [6], while dosage of mitoTEMPO was chosen based on the literature [13] and our preliminary study.

In some case, the cells were transfected with siRNA targeting OPA1 to knockdown OPA1 (NM_130835). SiRNA for OPA1 or scramble was purchased from GenePharma (Shanghai, China). The sequences for targeting human negative control are sense: 5′-UUCUCCGAACGUGUCACGUTT-3′, antisense: 5′-ACGUGACACGUUCGGAGAATT-3′. The sequences for targeting human OPA1 are sense: 5′-CCAUGUGGCCCUAUUUAAATT-3′, antisense: 5′-UUUAAAUAGGGCCACAUGGTT-3′. Lipo8000™ Transfection Reagent (Beyotime) was used to transfect siRNA into the cells. After 12 h transfection, the cells were replaced with fresh media, and incubated for 24 h. 

### 2.16. Migration and Invasion Assay

Migration of HTR8 cells was measured using Boyden chamber with an 8-μm pore size (Corning) as described previously [6,14]. Briefly, the cells were seeded into the top chamber, and were allowed to migrate toward the bottom chamber containing culture media in which cortisol (10^−6^ M), CBX (10^−6^ M) and mitoTEMPO (10^−7^ M) were added. In some cases, scramble siRNA and OPA1 siRNA mixed with Lipo8000™ were added into the cells to knockdown OPA1. After 12 h-incubation, the culture media were changed into fresh media. After 24 h incubation, the underside of the membrane was stained with DAPI (Beyotime). The number of the cells on the membrane from 3 random fields at ×200 magnification was counted under the microscope. 

Invasion function was assessed by using 24-well BD Matrigel invasion chambers (BD Biosciences) [6]. Briefly, cells were seeded in the inserts and treated with cortisol (10^−6^ M), CBX (10^−6^ M) and mitoTEMPO (10^−7^ M) for 24 h, or scramble siRNA and OPA1 siRNA mixed with Lipo8000™ were added. Noninvaded cells on the top of the filter were scraped off, and the invaded cells were fixed and stained with DAPI for microscopic analysis. Invaded cells from 3 random fields at ×200 magnification were counted under the microscope.

### 2.17. Assay of Mitochondrial Oxygen Consumption

The mitochondrial oxygen consumption rate (OCR) was measured in a Seahorse XFe96 Flux Analyzer (Seahorse Biosciences, Agilent, Santa Clara, CA, USA). Briefly, the cells were seeded in XFe96 cell culture plate at a density of 5000/well. The cells were then treated with cortisol (10^−6^ M) in the presence or absence of CBX (10^−6^ M) for 24 h. In some cases, scramble siRNA and OPA1 siRNA mixed with Lipo8000™ were added into the cells to knockdown OPA1. After 12 h-incubation, the culture media were changed into fresh media and incubated for 24 h. Before the assay, cell culture media were changed to the XF assay media supplemented with 5 mM sodium pyruvate (Thermo Scientific, Waltham, MA, USA), 10 mM glucose (Thermo Scientific, Waltham, MA, USA), and 2 mM glutamine, and equilibrated in an incubator for 1 h. Then XFe96 extracellular flux assay plate was inserted into culture plate (One day before the test, the cartridge with XF calibrant was incubated in an incubator at 37 °C overnight to equilibrate). OCR was monitored through sequential injections of 1 μM oligomycin, 1 uM FCCP and 1 μM antimycin A (Seahorse XF Cell Mito Stress Test Kit, Agilent, Santa Clara, CA, USA). The values were obtained and normalized by protein content (μg) per well. The mean values from quadruplicate wells were displayed as the OCR (pmol O_2_/min/μg protein). The parameters of mitochondrial activity including basal respiration, ATP production, maximum respiration and spare respiratory capacity were also determined.

### 2.18. Determination of OPA1 Gene Promoter Activity

Human optic atrophy 1 (OPA1) promoter (−2000 bp) were cloned into pGL3-luciferase reporter vector (GenePharma Corporation). HTR8 cells were transfected with the pGL3-luciferase reporter vector and pRL-TK-Renilla-luciferase plasmid (Promega Corp) using Lipo8000™ for 12 h. Then cells were treated with cortisol (10^−6^ M) with or without CBX (10^−6^ M) for 24 h. Luciferase assays were measured by Dual-Luciferase^®^ Reporter Assay System (Promega).

### 2.19. Human Samples

Ethics approval was obtained from the Ethics Committee of Medical Research of Xiangya Hospital Central South University. Informed consent was obtained from all participants accordingly. Placenta tissues were obtained from pregnant women with normotension at term (*n* = 24; 37–40 weeks) and PE subjects (*n* = 24; 36–40 weeks) undergoing elective cesarean section. PE was defined as pregnancy-induced hypertension (blood pressure ≥ 140/90 mm Hg) and proteinuria (≥0.3 g/24 h) in women who were normotensive before pregnancy and had no other underlying clinical problems. In the present study, late onset PE patients were enrolled. The Clinical characteristics of the pregnant woman enrolled in this study are shown in Table S3.

### 2.20. Statistical Analysis

GraphPad-Prism7 was used for graphic presentation and statistical analysis. Results are represented as the mean ± SD. Normal distribution was assessed by Shapiro–Wilk test. Statistical significance was determined according to sample distribution and homogeneity of variance. Statistical comparisons between two groups (animal experiments) were determined by two-tailed Student’s *t* test. In some cases, One-way ANOVA followed by Bonferroni’s post hoc test was performed for comparisons among multiple groups. *p* < 0.05 was considered statistically significant.

## 3. Results

### 3.1. Many Molecular Pathways Are Affected in CBX-Induced PE-Like Model by Omics Analysis

CBX treatment could lead to PE-like features including elevated MAP, systolic blood pressure (SBP), kidney damage, increased circulatory sFlt-1 and sEng levels and impaired placental blood flow in pregnant rats (Supplemental Figure S1), which is similar to our previous study [6]. Moreover, we showed that CBX treatment did not affect blood pressure in nonpregnant female rats compared with those treated with saline (Supplemental Figure S2). No obvious morphologic change in glomeruli and proteinuria were detected in non-pregnant rats with CBX treatment. 

**Figure 1:**
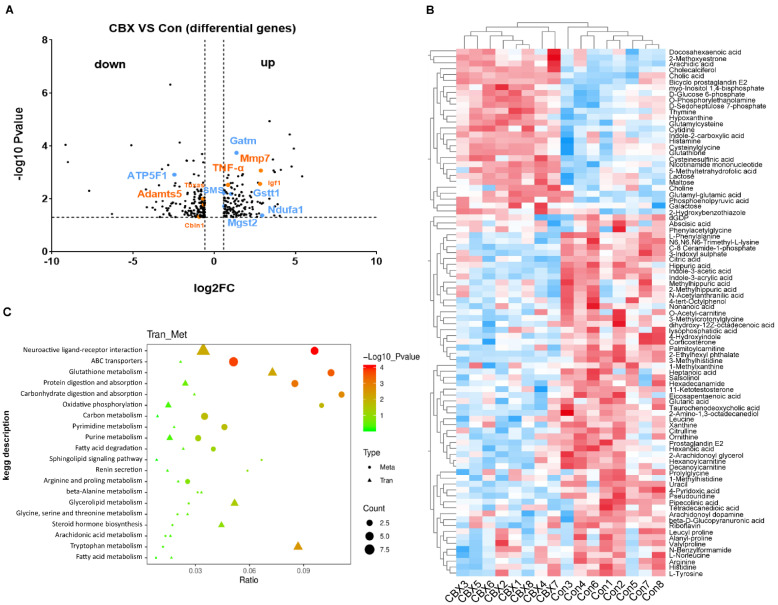
Transcriptomics and metabolomics of the placentas in rat PE−like model. Pregnant rats were administrated with CBX or saline from GD 7.5 to GD 17.5. The placentas were collected on GD 20.5 for RNA-seq and untargeted metabolomics. (**A**) volcano plot of differential genes in RNA-Seq between PE-like rats and controls. Orange: genes associated with PE. Blue: genes in OXPHOS, TCA cycle and glutathione metabolism. *n* = 5 in each group. (**B**) Cluster heat map of the differential metabolites. *n* = 8 in each group. (**C**) KEGG enrichment analysis combined with transcriptomics and metabolomics. Con: control. Tran: transcriptomics; Met: metabolomics.

RNA-seq was performed to investigate transcriptomics in the placentas. Heatmap hierarchical clustering clearly identified a different signature between CBX-induced PE-like group and control group (Supplemental Figure S3A). There were 145 up-regulated and 176 down-regulated genes in PE-like groups compared to the control group. Among them, there were genes in OXPHOS, tricarboxylic acid (TCA) cycle and glutathione metabolism (Figure 1A, blue marked). Of note, many genes including TNF-α, MMP7, ADAMts5, Igf1 etc. (Figure 1A, orange marked) have been reported to be associated with PE development [15,16,17,18]. KEGG pathway GO enrichment analysis showed that amino acid metabolism, sphingolipid pathway, mitochondrial functionalities, oxidative phosphorylation (OXPHOS), carbon metabolism and glutathione metabolism were affected in a PE-like model (Supplemental Figure S3B,C, Figure 1C). 

Metabolomics is a systematic approach that comprehensively analyzes endogenous metabolites [19]. Metabolites are downstream products of the genome and proteome, which are highly relevant to the function and phenotype of biological systems [20]. Untargeted metabolomic analysis was then performed to obtain the metabolomic profile in placentas of the PE-like model. There were 89 differential metabolites between the PE-like group and control group (Figure 1B). Among them, 61 metabolites were decreased, while the other 28 metabolites were increased. KEGG enrichment analysis showed that many pathways including phenylalanine metabolism, arginine biosynthesis, TCA cycle, galactose metabolism and glutathione metabolism were affected in the placentas of the PE-like model compared to controls (Supplemental Figure S3D). 

Figure 1C shows the integrative KEGG enrichment of transcriptome and metabolomics in the CBX-induced PE-like model. Neuroactive ligand-receptor interaction, OXPHOS, carbon metabolism, glutathione metabolism and purine metabolism, were enriched in both of transcriptome and metabolomics. Thus, these data indicate that core changes occur in OXPHOS, oxidative and antioxidative balance, and energy metabolism in CBX-induced PE-like model. 

### 3.2. Oxidative Phosphorylation (OXPHOS) and Mitochondrial Functions Are Impaired in the Placentas of CBX-Induced PE-Like Model

**Figure 2:**
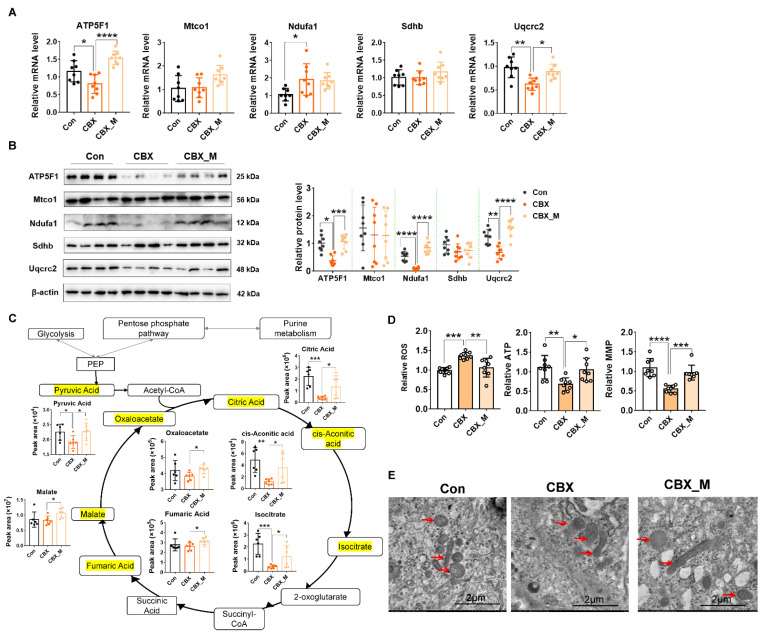
OXPHOS and mitochondrial functions are impaired in the placentas of PE-like model and mitoTEMPO treatment reverses them. Pregnant rats were administrated with CBX or saline from GD 7.5 to GD 17.5. The rats were sacrificed on GD 20.5, and the placentas were collected for analysis of mRNA and protein expression of various genes, targeted metabolomics and morphology. (**A**) the mRNA of the gene in OXPHOS electron transport chain components. (**B**) the protein levels of the gene in OXPHOS electron transport chain components. Left panel: representative images of Western blotting. Right panel: cumulative data of each protein expression level. (**C**) targeted metabolomics analysis of central carbon metabolism. Statistical chart shows peak area abundance of significantly altered metabolites in TCA cycle. (**D**) mitochondrial function assay of ROS, ATP and MMP. (**E**) electron microscope analysis of placental tissue. Representative images of electron microscope (10,000×). Red arrow: mitochondria. * *p*  <  0.05, ** *p*  <  0.01, *** *p*  <  0.001, **** *p*  <  0.0001. Con: control.

The above data indicate that placental OXPHOS is one of core changes in the CBX-induced PE-like model. We therefore examined the gene program in the OXPHOS pathway by using Q-PCR. As shown in Figure 2A, Ndufa1 mRNA levels (in complex I) were increased whereas mRNA levels of Uqcrc2 (in complex III), and ATP5F1 (in complex V) were decreased in the PE-like group compared with the control group. The mRNA levels of Sdhb (in complex II) and Mtco1 (in complex IV) were not significantly altered in the PE-like group. Western blotting analysis showed that Ndufa1, Uqcrc2 and ATP5F1 protein levels were significantly downregulated. In contrast, Sdhb and Mtco1 expression was not significantly altered in the CBX group compared with the control (Figure 2B), suggesting that mitochondrial electron transport chain components I, III and V are affected in the CBX-induced PE-like model. 

Central carbon metabolism is associated with OXPHOS function. Targeted metabolomics analysis of central carbon metabolism was performed in order to reveal the phenotype related to mitochondrial function. Among 31 metabolites, the differential metabolites with statistical significance were pyruvic acid, citric acid, isocitrate and cis-aconitic acid, the metabolites in TCA cycles. They were significantly decreased in the PE-like model (Figure 2C) compared with controls, confirming that energy metabolism related to the OXPHOS function is impaired in the placentas of this PE-like model. 

We further examined mitochondrial function and morphology. It showed that mitochondrial ROS production was significantly increased, whilst ATP level and MMP were significantly decreased in the PE-like group compared with controls (Figure 2D). Transmission electron microscope analysis showed that the number of mitochondria was reduced, and the remaining mitochondria exhibited aberrant cristae and a significant alteration in the matrix structure and the membranes in placentas of PE-like models (Figure 2E). 

### 3.3. MitoTEMPO Significantly Alleviates PE-Like Feature and Improves Placental Blood Flow and Placentation in CBX-Induced PE-Like Model

**Figure 3:**
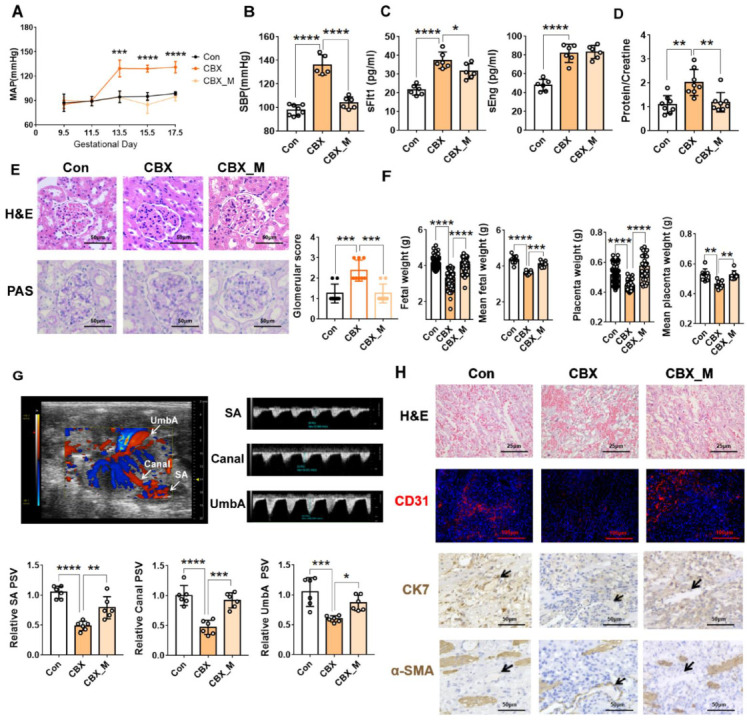
MitoTEMPO alleviates PE-like feature and improves placental blood flow and placentation in PE-like model. Pregnant rats were administrated with CBX, CBX combined with mitoTEMPO or saline from GD 7.5 to GD 17.5. Urine was collected from GD 18.5 to GD 19.5. After determination of arterial BP, the rats were sacrificed on GD 20.5 for collection of blood and tissues. (**A**) MAP measured from GD 9.5 until GD 17.5. (**B**) SBP measured on GD 20.5. (**C**) the circulatory sFlt1 and sEng levels in the rat model. (**D**) protein/creatinine (mg/mg) in urine in the rat model. (**E**) morphology of glomeruli stained by H&E and PAS. **Left panel**: the representative images (400×). **Right panel**: histopathological score of glomerular pathology. (**F**) fetal and placental weight from 8 dams (each group) measured on GD 20.5. It represents as individual fetal or placental weight and mean fetal and placental weight from each dam. (**G**) Doppler ultrasonography. **Upper panel**: the representative images of SA in implantation sites, canal in placentas and fetal UmbA visualized by ultrasound biomicroscopy. **Lower panel**: cumulative data of the PSV of SA, Canal and UmbA. (**H**) placental morphology, placental vasculature network and SP remodeling in the rat model. Representative H&E images of labyrinth zone (400×), representative immunofluorescence image of CD31 (100×), representative image of CK7 staining in SA, and representative image of α-SMA staining in SA (200×). * *p*  <  0.05, ** *p*  <  0.01, *** *p*  <  0.001, **** *p*  <  0.0001. Con: control; CBX_M: CBX combined with mitoTEMPO treatment.

Many studies have demonstrated that excess mitochondrial ROS would further impair OXPHOS, which constitutes a feed-forward loop in mitochondrial dysfunction [21,22]. We therefore hypothesized that excess mitochondrial ROS is the key event in CBX-induced PE. Mitochondrial superoxide scavenger mitoTEMPO was then used to block mitochondrial superoxide production as described previously [8,23,24]. As shown in Figure 3A–F, mitoTEMPO treatment significantly alleviated PE-like features as evidenced by reduced MAP and SBP and decreased sFlt-1 level, and by improvement of the protein/creatinine in urine and renal injury in CBX-induced PE-like rats. Moreover, fetal weight was also improved. MitoTEMPO alone had no significant effect on BP, renal morphology, and fetal and placental growth (Supplemental Figure S4). 

We examined the effects of mitoTEMPO on placental development, spiral artery remodeling and placental blood flow in the CBX-induced PE-like model. Increased PSV of SA, canal and UmbA was found in the placentas of the PE-like model with mitoTEMPO treatment (Figure 3G). H&E staining showed that organization of the labyrinth was improved and CD31 staining showed branching of vasculature was increased (Figure 3H). Immunocytochemistry showed that α-SMA staining in spiral artery wall was reduced whilst trophoblasts surrounding it were increased after mitoTEMPO treatment (Figure 3H). Given that PE development is attributed to abnormal placentation and impaired remodeling of the spiral artery, the above data indicate that mitoTEMPO treatment prevents 11β-HSD2-induced abnormal placentation and impairment in spiral artery remodeling, thereby alleviating PE-like features. 

### 3.4. MitoTEMPO Rescues Impaired Molecular Pathways and Decreased 11β-HSD2 Expression and Improves Mitochondrial Function in CBX-Induced PE-Like Model

Next, we investigated whether mitoTEMPO treatment improves impaired signaling pathways in placentas of the CBX-induced PE-like model. RNA-seq analysis showed mitoTEMPO treatment results in changed expression of many genes in the PE model (Supplemental Figure S5A). Enrichment analysis showed that the enriched pathways in PE-like rats with mitoTEMPO treatment group were basically related to mitochondria (Supplemental Figure S5D). Untargeted metabolomics also showed that a few metabolites, such as citric acid and prostaglandin E2, were reversed by mitoTEMPO (Supplemental Figure S5B). Integrative KEGG analysis of transcriptome and metabolomics showed that many pathways including OXPHOS, glutathione metabolism, glycolysis/gluconeogeneis, pyruvate metabolism, arginine and proline metabolism were enriched (Supplemental Figure S5C). The above data indicate that OXPHOS, oxidative and antioxidative balance, and energy metabolism were key pathways in response to mitoTEMPO treatment in the CBX-induced PE-like model. 

It is known that the changes in glutathione metabolism are associated with oxidative stress and mitochondrial function [25,26]. This pathway was enriched in both the PE group and PE with mitoTEMPO treatment group. Q-PCR confirmed that increased Gstt1, Mgst2, Gatm and SMS mRNA levels were significantly changed in the PE-like group, which was reversed by mitoTEMPO treatment (Supplemental Figure S6A), suggesting that oxidative and antioxidative balance is improved by mitoTEMPO. In addition, our previous study shows that 11β-HSD2 expression is inhibited in the PE-like model [6]. In the present study, we found that decreased 11β-HSD2 expression was partly reversed by mitoTEMPO treatment in PE-like rats (Supplemental Figure S6B), suggesting that excess ROS is involved in downregulation of 11β-HSD2. 

MitoTEMPO treatment improved the gene program of OXPHOS and mitochondrial function and morphology. As shown in Figure 2A,B, changed mRNA and protein levels of the genes in OXPHOS were reversed by mitoTEMPO treatment in CBX-induced PE-like rats. Targeted metabolomics analysis of central carbon metabolism showed that mitoTEMPO treatment not only reversed the levels of citric acid, isocitrate and cis-aconitic acid, but also increased the levels of most metabolites in TCA cycles in PE rats (Figure 2C). Figure 2D shows mitoTEMPO treatment significantly improved MMP and ATP production and reduced ROS production in PE-like rats. Transmission electron microscope analysis showed mitochondrial morphology was improved upon mitoTEMPO treatment (Figure 2E). These data suggest that excess ROS plays a central role in mitochondrial dysfunction. 

### 3.5. MtDNA Maintenance and Mitochondrial Dynamics Are Disrupted in the Placentas of CBX-Induced PE-Like Model and mitoTEMPO Treatment Rescues Them

**Figure 4:**
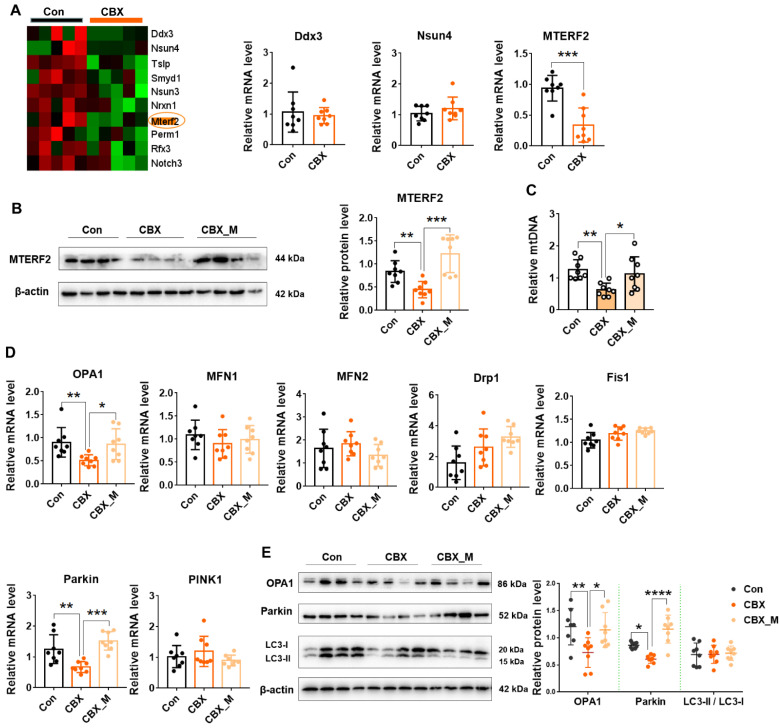
MitoTEMPO improves MtDNA stability and mitochondrial dynamics in the placentas of PE-like model. Pregnant rats were administrated with CBX, CBX combined with mitoTEMPO or saline from GD 7.5 to GD 17.5. The rats were sacrificed on GD 20.5 for collection of blood and placental tissues. (**A**) the transcriptional levels of the genes that control mtDNA maintenance. **Left panel**: heatmap of the genes related to mtDNA maintenance in RNA-seq. **Right panel**: cumulative data of Q-PCR analysis. (**B**) MTERF2 protein expression level. (**C**) mtDNA copy number. (**D**) the mRNA levels of mitochondrial dynamic genes. (**E**) protein levels of OPA1, Parkin and LC3. **Left panel**: representative images of Western blotting. **Right panel**: cumulative data of each protein expression level. * *p*  <  0.05, ** *p*  <  0.01, *** *p*  <  0.001, **** *p*  <  0.0001. Con: control; CBX_M: CBX combined with mitoTEMPO treatment.

We intended to further investigate the molecular mechanisms responsible for mitochondrial dysfunction in the placentas of PE-like model. Emerging evidence indicates that mtDNA content is a biomarker of mitochondrial function because mtDNA copy number is essential for OXPHOS function [27,28,29,30]. Of note, RNA-seq showed that the levels of a few genes that regulate mtDNA maintenance such as Ddx3, Nsun4 and MTERF2 were changed (Figure 4A). Among them, MTERF2 mRNA and protein level were significantly downregulated in the PE-like model (Figure 4B). MtDNA content was significantly decreased in PE-like rats compared with controls (Figure 4C). MitoTEMPO treatment reversed METRF2 expression and mtDNA content in placenta of the PE-like model (Figure 4B,C). 

Given that mitochondria undergo autophagy and fusion/fission from time to time in order to maintain their proper functions [31], we then investigated whether mitochondrial dysfunction is associated with impaired mitochondrial dynamics. The mRNA levels of Mfn1, Mfn2, Drp1, Fis1, PINK were not significantly changed in the PE-like group (Figure 4D). The mRNA levels of OPA1, a protein of inner membrane responsible for mitochondrial fusion, and Parkin, an autophagy-related molecule, were significantly reduced in the CBX-induced PE model compared with controls. Consistently, OPA1 and Parkin protein levels were also significantly reduced in the PE-like group. MitoTEMPO treatment significantly reversed downregulated OPA1 and Parkin expression. However, LC3-II/LC3-I levels were not significantly altered in the PE-like model (Figure 4E). The above data indicate that mtDNA instability and disruption of mitochondrial dynamics are associated with mitochondrial dysfunction and excess ROS is a key factor that leads to mtDNA instability and disruption of mitochondrial dynamics. 

### 3.6. 11β- HSD2 Dysfunction Impairs Mitochondrial Function in Cultured Human EVTs

**Figure 5:**
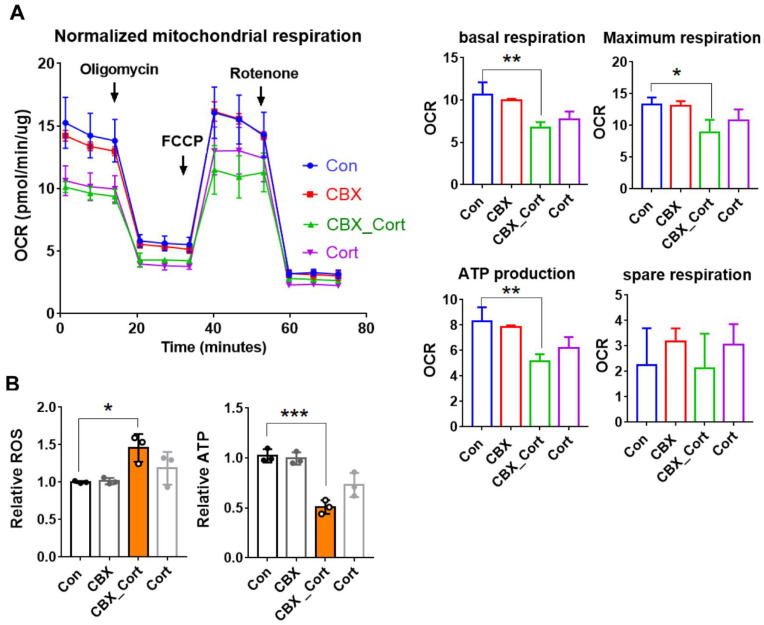
The effects of 11β-HSD2 dysfunction on mitochondrial function and MtDNA content in EVTs. HTR8 cells were treated with cortisol (10^−6^ M) in the presence and absence of CBX (10^−6^ M) for 24–48 h, and then the cells were used for OCR analysis, or harvested for mitochondria isolation and Q-PCR analysis. (**A**) Seahorse mitochondrial stress assay. **Left panel**: representative traces of OCR. **Right panel**: cumulative data of basal respiration, maximal respiration, ATP production and spare respiratory capacity. (**B**) mitochondrial function assay of ROS and ATP production. *n* = 3 independent cultures. * *p* < 0.05, ** *p*  <  0.01, *** *p*  <  0.001 Con: control; Cort: cortisol, CBX_Cort: CBX combined with cortisol.

We showed that mitoTEMPO treatment prevents 11β-HSD2-induced abnormal placentation and impairment in spiral artery remodeling in pregnant rats. It is known that proper EVT functions are crucial for placental development and spiral artery remodeling [32]. Our previous study has shown that 11β-HSD2 dysfunction leads to excess GC accumulation and subsequently results in impaired invasion and migration of cultured human EVTs [6]. However, the molecular mechanisms underlying impaired functions of EVTs caused by 11β-HSD2 dysfunction remain largely unknown. As mentioned, major processes are similar in spiral artery remodeling and placentation between rodents and humans. We therefore investigated the roles of mitochondria in EVT functions in cultured HTR8, a human EVT cell line. First, we studied whether mitochondrial function is affected by 11β-HSD2 dysfunction in HTR8 cells. Consistent with the animal study, OCR analysis showed that basal respiration rate and maximal respiration rate were suppressed by cortisol combined with 11β-HSD2 inhibitor treatment (Figure 5A). ATP production was reduced upon to CBX combined with cortisol treatment. Mitochondrial function analysis showed that ATP production was inhibited whilst ROS production was promoted by cortisol combined with 11β-HSD2 inhibitor treatment (Figure 5B). These data suggest that 11β-HSD2 dysfunction leads to increased GC availability locally, and subsequently causes mitochondrial dysfunction. 

### 3.7. MitoTEMPO Treatment Improves Impaired Migration and Invasion Function, MtDNA Maintenance and Mitochondria Dynamic Caused by 11β-HSD2 Dysfunction in Human EVTs

**Figure 6:**
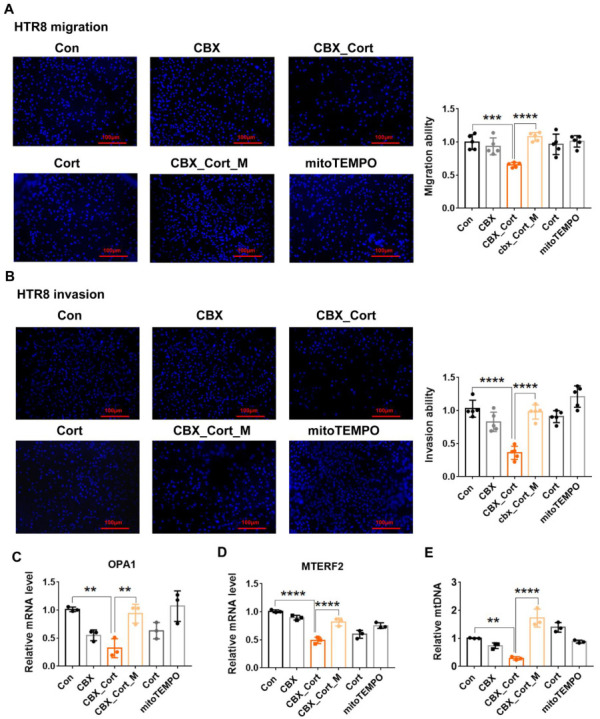
MitoTEMPO treatment improves impaired migration and invasion function, MtDNA maintenance and mitochondria dynamic caused by 11β-HSD2 dysfunction in EVTs. HTR8 cells were treated with cortisol (10^−6^ M), CBX (10^−6^ M), mitoTEMPO (10^−7^ M) or their combination for 24 to 48 h. The cells were then used for the migration and invasion analysis as described in Methods. In some cases, cells were harvested for Q-PCR analysis. (**A**) the migration function analysis. **Left panel**: the fluorescence microscopic images show that the cells moved to the underside of the membrane (100 ×. **Right panel**: histogram shows the cumulative data of migration function. (**B**) the invasion function analysis. **Left panel**: the fluorescence microscopic images show that the cells moved to the underside of the membrane (100×). **Right panel**: histogram shows the cumulative data of invasion function. (**C**,**D**) the mRNA levels of MTERF2 and OPA1. (**E**) mtDNA copy number. *n* = 3 independent cultures. ** *p* < 0.01, *** *p* < 0.001, **** *p* < 0.0001. Con: control; Cort: cortisol, CBX_Cort: CBX combined with cortisol. CBX_Cort_M: CBX combined with cortisol and mitoTEMPO treatment.

Next, we explored whether excess ROS plays a critical role in 11β-HSD2 dysfunction induced impairment of EVT functions. As shown in Figure 6A,B, CBX combined with cortisol treatment suppressed migration and invasion of HTR8 cells. MitoTEMPO treatment significantly increased migration and invasion function in the cells with CBX combined with cortisol treatment.

We then examined whether mitochondrial dynamics and mtDNA stability are affected by 11β-HSD2 dysfunction in HTR8 cells. We mainly focused on OPA1 and MTERF2 expression in mitochondrial dynamics. As shown in Figure 6C–E, CBX combined with cortisol treatment inhibited OPA1 and MTERF2 mRNA expression. MitoTEMPO treatment significantly increased OPA1 and MTERF2 expression. CBX combined with cortisol treatment resulted in a decrease in mtDNA content, which could be reversed by mitoTEMPO treatment. The above data indicate that excess ROS play a key role in mitochondrial dysfunction, mtDNA instability and impaired mitochondrial dynamics caused by 11β-HSD2 dysfunction, which is consistent with the findings in animal model. 

### 3.8. OPA1 Is One of Key Factors That Mediate Mitochondrial Dysfunction and Impaired Migration and Invasion Function Caused by 11β-HSD2 Dysregulation

**Figure 7:**
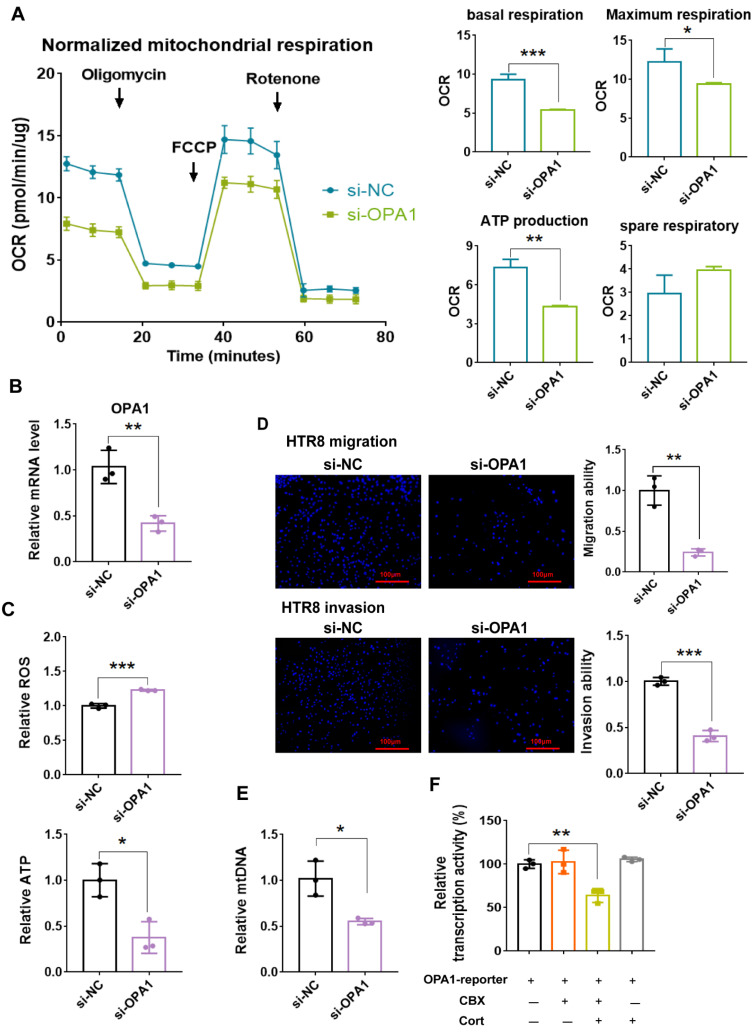
OPA1 downregulation leads to mitochondrial dysfunction and impaired migration and invasion function in EVTs and OPA1 transcription is regulated by 11β-HSD2 in EVTs. HTR8 cells were transfected with scramble siRNA (si-NC) and OPA1 siRNA (si-OPA1) for 24 h, respectively. Then the cells were used for OCR analysis or harvested for mitochondria isolation and Western blotting and Q-PCR analysis. In some cases, the cells were used for migration and invasion analysis as described in Methods. (**A**) Seahorse mitochondrial stress assay. **Left panel**: representative traces of OCR. **Right panel**: cumulative data of basal respiration, maximal respiration, ATP production and spare respiratory capacity. (**B**) validation of OPA1 knockdown at protein level by Western blotting. (**C**) mitochondrial function assay of ROS and ATP production. (**D**) the migration and invasion function in HTR8 cells. Upper left panel: the fluorescence microscopic images show that the cells moved to the underside of the membrane (100×). Upper right panel: histogram shows the cumulative data of migration function. Lower left panel: the fluorescence microscopic images show that the cells moved to the underside of the membrane (100×). Lower right panel: histogram shows the cumulative data of invasion function. (**E**) mtDNA copy number. (**F**) transcriptional activity of OPA1 promoter. HTR8 cells were transfected with pGL3-luciferase reporter containing OPA1 promoter and combination with pRL-TK-Renilla-luciferase plasmid for 12 h. Cells were then treated with cortisol (10^−6^ M) combined with CBX (10^−6^ M) for 24 h. Luciferase assays were performed using the dual luciferase assay kit. *n* = 3 independent cultures. * *p*  <  0.05, ** *p*  <  0.01, *** *p*  <  0.001.

Some studies have demonstrated that OPA1, a protein of inner membrane responsible for mitochondrial fusion, is critical for maintenance of mitochondrial function [33,34,35]. Since OPA1 downregulation was found in both the intact animal model and cultured cell model upon 11β-HSD2 dysfunction, we sought to investigate the roles of OPA1 in maintenance of mitochondrial function as well as invasion and migration function in HTR8 cells. As shown in Figure 7B, OPA1 siRNA led to reduced OPA1 expression. Knockdown of OPA1 expression inhibited the basal respiration and maximal respiration, and decreased ATP production (Figure 7A). OPA1 knockdown inhibited ATP production whilst promoting ROS production (Figure 7C) and led to a decrease in mtDNA content (Figure 7D). A significant decrease in migration and invasion occurred in the cells with OPA1 knockdown compared with control siRNA (Figure 7E).

We then investigated whether OPA1 transcription is controlled by 11β-HSD2 in trophoblasts. It was found that cortisol combined with CBX treatment suppressed the transcriptional activity of OPA1 promoter in HTR8 cells (Figure 7F), suggesting that 11β-HSD2 regulates OPA1 expression at transcriptional level. 

**Figure 8:**
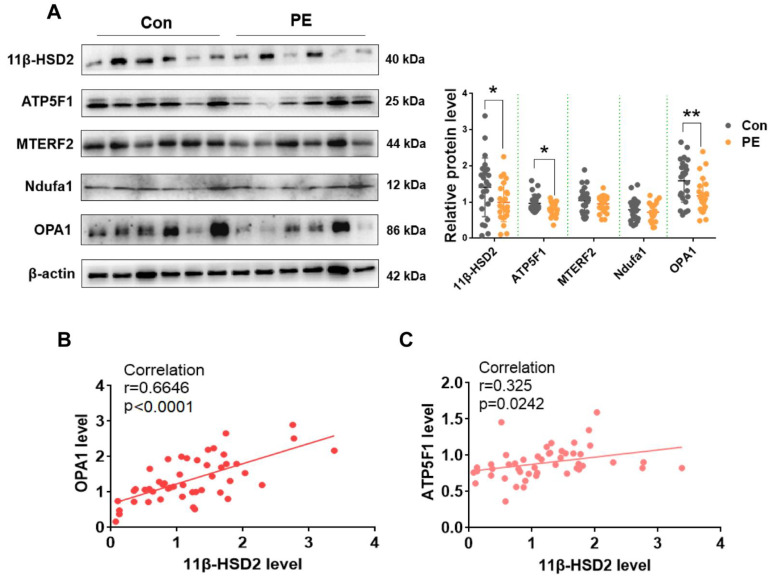
The correlation analysis of 11β-HSD2 level with OPA1, MTERF2, Ndufa1 and ATP5F1 levels in placentas of PE patients. (**A**) the expression levels of 11β-HSD2, OPA1, MTERF2, Ndufa1 and ATP5F1 in normotension (Con) and PE placentas. **Left panel**: representative images of Western blotting. **Right panel**: cumulative data of each protein expression level. (**B**) correlation analysis of grayscale value between 11β-HSD2 and OPA1. (**C**) correlation analysis of grayscale value between11β-HSD2 and ATP5F1. *n* = 24 in each group. * *p*  <  0.05, ** *p*  <  0.01.

We subsequently analyzed the correlation of 11β-HSD2 level with OPA1, ATP5bp, MTERF2 and Ndufa1 levels. It was found that OPA1 and ATP5F1 levels were significantly decreased in PE placentas (Figure 8). Positive correlations were found between OPA1 and 11β-HSD2 as well as ATP5F1 and 11β-HSD2, suggesting that OPA1 and ATP5F1 expression levels are associated with 11β-HSD2 level in human placentas. 

Placental 11β-HSD2 dysregulation leads to excess GCs accumulation, and subsequently results in reduced OPA1 expression and mtDNA stability, leading to mitochondrial dysfunction, which manifests as excess ROS, abnormal OXPHOS function and TCA cycles. Excess ROS further promotes OPA downregulation and mtDNA instability. Mitochondrial dysfunction leads to impaired invasion and migration function of EVTs, subsequently causing abnormal placentation. Elimination of excess ROS improves OXPHOS function, OPA1 expression and mtDNA maintenance, and subsequently alleviates PE-like features. 

## 4. Discussion

As mentioned, placenta is the principal contributor to PE development. Several studies have demonstrated transcriptome and metabolomic profiles in the placentas of PE patients in order to present the molecular network in PE placentas [36,37,38,39]. The present study has revealed that the molecular pathways such as sphingolipid pathway, OXPHOS, carbon metabolism, glutathione metabolism and mtDNA maintenance were affected in the placentas of a CBX-induced PE-like model. Interestingly, these molecular pathways are reported in the placentas of PE patients [36,37,38,39,40,41,42,43]. In addition, we found that TNF-α, MMP7, ADAMts5 mRNA levels were increased in the PE-like model, which is consistent with prior studies of human PE placentas where they are implicated to be involved in PE pathogenesis [15,16,17,18]. These results indicate that placental 11β-HSD2 is a key factor in PE development, and the 11β-HSD2 dysfunction-induced PE-like rat model resembles the characteristics of PE in humans. 

More recently, the association of placental mitochondrial dysfunction with PE has received much attention. Many studies have reported that mitochondrial dysfunction occurs in the placentas of PE patients [44,45,46]. Placental mitochondrial abnormalities are also reported in some PE-like animal models including reduced uterine perfusion pressure (RUPP) rat and mouse models and L-NAME rat model [8,47,48,49,50]. The present study strongly indicated that mitochondrial dysfunction was one of key events in PE development associated with 11β-HSD2 dysfunction: (1) unbiased omics analysis demonstrated that the core changes were basically linked to mitochondria; (2) mitochondrial function analysis showed abnormalities in ROS and ATP production and MMT; (3) abnormal morphology of mitochondria was presented; (4) targeted metabolomics of central carbon metabolism showed that the metabolites associated with mitochondrial energy metabolism were significantly changed. Whether placental mitochondrial dysfunction is a causative factor or just collateral damage in the development of PE remains to be elucidated. In the present study, we revealed that improvement of mitochondrial function by mitoTEMPO treatment alleviated the hallmark of PE-like features and improved the key pathological changes in placentas linked to PE development including placental morphology and spiral artery remodeling. Our data indicate that mitochondrial dysfunction acts a critical link to PE development. 

The present study has demonstrated that excess mitochondrial ROS production plays central roles in the development of PE caused by 11β-HSD2 dysfunction, and causes mitochondrial dysfunction, thereby leading to impaired functions of trophoblasts and abnormal placentation. Overproduction of ROS contributes to multiple organ injury by leading to cytosolic calcium overload, energy depletion, apoptosis/necrosis, and inflammation [51,52,53,54,55]. Emerging evidence indicates that about 90% of ROS are generated in mitochondria [56]. Mitochondrial ROS are primarily produced at complexes I and III of the electron transport chain when electrons derived from either NADH or FADH2 react with O2 [21,57]. Interestingly, we did find that OXPHOS complexes I and complex III were affected in the placentas of the PE-like model. The burst of ROS production causes direct oxidative damage to mitochondrial proteins, thereby impairing OXPHOS and leading to an increase in mitochondrial membrane permeability [58]. More recently, it has been demonstrated that mitochondrial ROS can disrupt mtDNA maintenance, thereby leading to mitochondrial dysfunction [28,29,30]. Of note, we provided convincing evidence that excess mitochondrial ROS production is central for mitochondrial dysfunction: (1) mitochondria-targeted antioxidant mitoTEMPO significantly improved OXPHOS function, reduced mitochondrial membrane permeability and reversed mtDNA copy numbers; (2) mitoTEMPO significantly improved mitochondrial energy metabolism; (3) mitoTEMPO improved impaired respiratory rates caused by 11β-HSD2 dysfunction in cultured EVTs. As mentioned, our study suggested that mitochondrial dysfunction is a key event during PE development. In fact, we provided evidence that excess ROS plays crucial roles in the development of PE associated with 11β-HSD2 dysfunction by showing that mitoTEMPO alleviated PE-features in pregnant rats, improving spiral artery remodeling, placental morphology and blood flow. Moreover, mitoTEMPO reversed the impaired molecular pathways caused by 11β-HSD2 dysfunction. Interestingly, excess ROS contributed to down-regulation of 11β-HSD2 expression in the placentas in the CBX-induced PE-like model. This suggests that 11β-HSD2 downregulation results in mitochondrial ROS accumulation, and the latter then further inhibits 11β-HSD2 expression. Elimination of excess ROS production would disrupt the above feed-forward loop. 

MtDNA encodes 13 protein subunits of the electron transport chain complexes and a set of transfer and ribosomal RNAs; thus, mitochondrial function is extremely dependent on functional mtDNA [59,60]. Maintenance of mtDNA stability is critical for proper OXPHOS function [27,59]. Some studies have shown that mtDNA content is reduced and OXPHOS components are impaired in the placenta of PE patients [37,61,62]. In the present study, we demonstrated that reduced mtDNA contributed to OXPHOS function. A few studies have reported that glucocorticoids can directly regulate mtDNA content [63,64,65,66]. Consistently, we showed that 11β-HSD2 dysfunction caused excessive GCs locally, and subsequently suppressed mtDNA content. Moreover, our study indicates that reduced mtDNA content is associated with excess mitochondrial ROS in the placentas by showing that mitoTEMPO treatment increased mtDNA content in the placentas. More recently, Zhao et al. [28] have demonstrated that mitochondrial ROS disrupts mtDNA stability by affecting mitochondrial transcriptional factor A (TFAM) level. However, we found that MTERF2, a regulator of mtDNA transcriptional machinery, was affected in the placenta of the CBX-induced PE-like model, and MTERF2 expression was reversed by elimination of excess ROS. Some studies have demonstrated that mtDNA stability is regulated by MTERF [67,68]. Thus, it might suggested that mtDNA instability caused by 11β-HSD2 dysfunction is associated with MTERF2 level. 

Mitochondrial dynamics are critical in maintaining mitochondrial homeostasis and functions. The degree to which mitochondria are networked results from a dynamic equilibrium between fusion and fission [69,70]. In the present study, we found that OPA1, a constituent of mitochondrial fusion machinery, was significantly downregulated in the placentas of 11β-HSD2 dysfunction-induced PE-like model and cultured EVTs with 11β-HSD2 dysfunction. OPA1 is in mitochondrial inner membranes and involved in mitochondrial fusion, thereby playing an important role in maintaining mitochondrial homeostasis and functions [33]. Some studies have demonstrated that OPA1 level is critical for respiratory chain function and ATP production in various cells [34,35]. Consistently, we found that OPA1 was critical for maintenance of mitochondrial respiration and ATP production in EVTs. Moreover, our data have demonstrated that OPA1 is also involved in maintenance of mtDNA stability. Given that OPA1 can modulate ROS production, OPA1 regulation of mtDNA stability might be through ROS production. Interestingly, we found that mitoTEMPO treatment reversed decreased OPA1 expression caused by 11β-HSD2 dysfunction, suggesting that excess ROS play a key role in OPA1 expression. Our study also showed that excess ROS production inhibited mtDNA level. Together, this suggests that excess ROS, mtDNA instability and reduced OPA1 form a loop linked to mitochondrial dysfunction. 

There may be controversies about the effects of mitochondria targeted antioxidant on PE-like features in animal models. For instance, Vaka et al. [8] reported that mitoTEMPO and mitoQ10 treatment from GD14 to GD 19 can alleviate hypertension in RUPP (surgery on GD14) rat model of PE. However, Yang et al. [71] reported that mitoQ10 treatment increases PE risk when administrated in early pregnancy in RUPP mouse model. In the present study, mitoTEMPO was administrated from GD7.5 to GD20.5 in CBX-induced PE-like model. It is obvious that the different model used accounts for this controversy. The RUPP model is a widely used PE model; however, it is well known that this model is different from human PE. In the RUPP model, low blood perfusion of uterus leads to placenta ischemia, thereby leading to hypertension in the animals. In contrast, our 11β-HSD2dysfunction-induced PE-like model is close to the PE that begins from abnormal placentation. 

There are a number of limitations in the present study. For instance, some studies have proposed that sex differences in molecular networks occur in placentas with adverse pregnant outcomes [72,73,74,75]. However, we did not distinguish female and male placentas in our determinations. It would be of interest to investigate whether mitoTEMPO treatment has different effects on female and male fetal and placental growth and other pregnant outcomes in 11β-HSD2 dysfunction-induced PE-like model. Another limitation is that we only analyzed the correlation between 11β-HSD2 and OPA1 or ATP5F in the placentas of late-onset PE patients. It is now known that the mechanisms underlying early-onset PE and late-onset PE are different. It will be important to investigate the association of 11β-HSD2 and OPA1 in early-onset PE placentas in our future studies.

## 5. Conclusions

The present study has demonstrated that the molecular pathways related to mitochondrial functions in placentas are the core pathways linked to PE pathogenesis associated with 11β-HSD2 dysfunction. Mitochondrial dysfunction impairs trophoblast function and subsequently results in abnormal placentation, thereby leading to PE development. Excess ROS production caused by OXPHOS dysfunction further promotes mitochondrial dysfunction. OPA1 and mtDNA stability are the key factors that mediate mitochondrial dysfunction induced by 11β-HSD2 dysfunction in placenta. Our study provides strong evidence that placental mitochondrial ROS is a therapeutic target for PE.

## Data Availability

All of the data is contained within the article and the Supplementary Materials.

## Supplementary Materials

The following supporting information can be downloaded at: https://www.mdpi.com/article/10.3390/antiox11081505/s1. Figure S1: 11β-HSD2 inhibition leads to PE-like features in pregnant rats; Figure S2: The effects of CBX on blood pressure and renal morphology in nonpregnant rats; Figure S3: Heatmap and pathway enrichment of transcriptomics and metabolomics of the placentas in rat PE-like model; Figure S4: The effects of MitoTEMPO alone on blood pressure, renal morphology, and fetal and placental weight in pregnant rats; Figure S5: Transcriptomics and metabolomics of the placentas in the PE-like model with mitoTEMPO treatment; Figure S6: The expression level of the genes in glutathione metabolism pathway and 11β-HSD2 expression in the PE-like model with mitoTEMPO treatment; Table S1: The primers for Q-PCR; Table S2: Antibodies for western blotting and immunofluorescence; Table S3: Clinical characteristics of the pregnant woman enrolled in this study.
