# Supplementary material for: Mitochondria Targeted Antioxidant Significantly Alleviates Preeclampsia Caused by 11β-HSD2 Dysfunction via OPA1 and MtDNA Maintenance
Source: Antioxidants (Basel). 2022 Jul 31;11(8):1505. doi: 10.3390/antiox11081505 (PMC9404992; doi:10.3390/antiox11081505)
Supplement: Supplementary file 1 [file antioxidants-11-01505-s001.zip › antioxidants-1777240-supplementary.pdf]

Online supplement tables and figures

Table S1. The primers for Q-PCR

	Forward Primer (5' -> 3')	Reverse Primer (5' -> 3')
β -actin（Rat）	CGTAAAGACCTCTATGCCAACA	TAGGAGCCAGGGCAGTAATC
11β-HSD2（Rat）	GACTAATGTGAACCTCTGGGAG	TCAGTGCTCGGGGTAGAAGGTG
Ndufa1(Rat)	GGTTGGAGTGTGAGTAACGGT	TCCAGGCCCTTGGACACATAG
ATP5F1(Rat)	GTCCCGGGTGGTACTTTCTG	GGTAAGTGACCTCCAAGGCC
Gstt1(Rat)	CCAGTCTTTGAAGGGCGTCC	GGGCGCACAGTCGTGTAATG
Mgst2(Rat)	TTCAATCAAGTTTTTGCAACC	TCTTGGCAACATGAAAGTCC
Gatm(Rat)	GCCTCGAGACATCCTGATGG	GATCACAGGTGTTGGAGGGG
SMS(Rat)	GGATTGGTATTGCTGGACCT	CCAAATTAACATCCCCGCTG
TNF-α(Rat)	TGCCTCAGCCTCTTCTCATT	TGGTATGAAGTGGCAAATCG
MMP7(Rat)	GGTGTGGAGTGCCAGATGTT	ACCATCCGTCCAGTACTCAT
ADAMts5(Rat)	CCCAAATACGCAGGTGTCCT	ACACACGGAGTTGCTGTAGG
Sdhb(Rat)	GGAGGGCAAGCAACAGTATC	TTGTCTCCGTTCCACCAGTAA
Uqcrc2(Rat)	TGCAGCCTCAGGAACTTGAG	ACCGAAACCAACCTGAACCA
Mtco1(Rat)	CCCACTTTGCCATTATATTTGTAGG	TTTCATGTGGTGTAAGCATCTGG
MTERF2(Rat)	GACCTACGCCGAGGAGATTG	CGGAGTCTGTGAAGCCTTGT
Ddx3(Rat)	TTATACACGCCCAACTCC	GACGCCCATACTTTCCAT
Nsun4(Rat)	TCGGAGTTATTGGCGTTGCT	TCCATGCTGCAGACCAAGAG
OPA1(Rat)	TCACTGCGGGTACACCTGG	CTGACACCTTCCTATAGTGCTTG

		T
MFN1(Rat)	GCTGCATACAGACAGACAGCCT	GGTAATGACCTGTCTCAGGGCT
MFN2(Rat)	CACTACCACATCGGACACCCTA	GAACTTGTGTCTTGCATTTGGC
Drp1(Rat)	GAAGTGGTGCAGTGGAAATGAC	GTTTCTATTGGGAACCACTGCC
Fis1(Rat)	GCACGCAGTTTGAATACGCC	GCTGCTCCTCTTTGCTACCTT
Parkin(Rat)	GAGCTAAACCCACCTACCACAG	CATCCGGTTTGGAATTAAGACA
PINK1(Rat)	TGCAATGCCGCTGTGTATGA	TCTGCTCCCTTTGAGACGAC
RPS18(Rat)	TCTTCCACAGGAGGCCTACA	ACAGCAAAGGCCCAAAGACT
16SrRNA(Rat)	GGTGCAGCCGCTATTAAAGG	ATCATTTACGGGGGAAGGCG
β-actin(Human)	GGCACCCAGCACAATGAAG	CCGATCCACACGGAGTACTTG
B2M(Human)	TGTTCCTGCTGGGTAGCTCT	CCTCCATGATGCTGCTTACA
16SrRNA(Human)	GGTGCAGCCGCTATTAAAGG	ATCATTTACGGGGGAAGGCG
MTERF2(Human)	GAGGATGAAACCTATGTTGAAG	ACAGACATTGCTTCCGGGCAGC
OPA1(Human)	GGCTCTGCAGGCTCGTCTCAAGG	TTCCGCCAGTTGAACGCGTTTACC

Table S2. Antibodies for western blotting and immunofluorescence

Antibody	Manufactory	Catalog Number	Host / Isotype
11β-HSD2	Proteintech	14192-1-AP	Rabbit / IgG
ATP5F1	Proteintech	15999-1-AP	Rabbit / IgG

CD31	abcam	ab222783	Rabbit / IgG
LC3	Proteintech	14600-1-AP	Rabbit / IgG
Mtco1	Immunoway	YN0177	Rabbit / IgG
MTERF2	Immunoway	YT6815	Rabbit / IgG
Ndufa1	Boster	BA3676	Rabbit / IgG
OPA1	abcam	ab42364	Rabbit / IgG
Parkin	Proteintech	14060-1-AP	Rabbit / IgG
Sdhb	Proteintech	10620-1-AP	Rabbit / IgG
Uqcrc2	Proteintech	14742-1-AP	Rabbit / IgG
β-actin	Proteintech	20536-1-AP	Rabbit / IgG

Table S3. Clinical characteristics of the pregnant woman enrolled in this study

	Normotension (n=24)	PE (n=24)	P value
Maternal age (years)	32.25±4.376	33.29±4.309	0.4103
BMI (kg/m^2)	25.96±3.219	30.05±4.352	0.0006
Gestational age (wk)	39.49±0.6810	37.39±1.363	＜0.0001
Systolic blood pressure (mmHg)	112.8±13.24	153.5±13.33	＜0.0001
Proteinuria (g/24h)	NA	3.197±2.713	NA
Infant birth weight (g)	3286±261.3	2510±608.1	＜0.0001

Statistical analysis was performed by two-tailed Student’s t test.

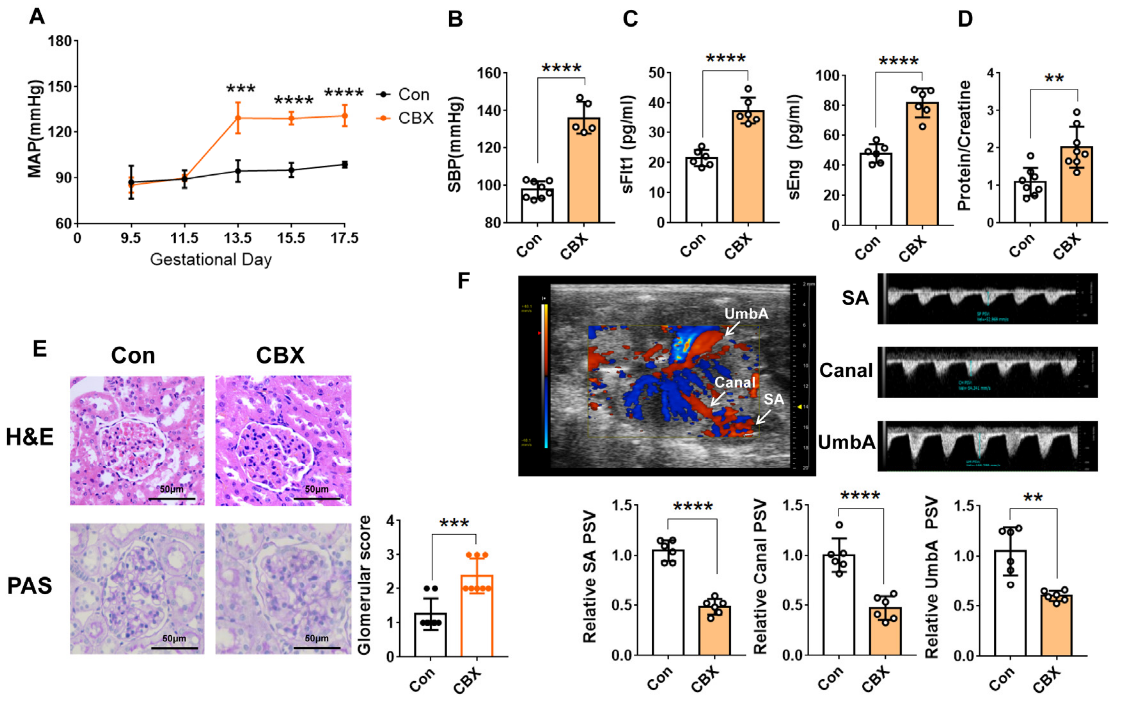

Figure S1. 11β-HSD2 inhibition leads to PE-like features in pregnant rats. Pregnant rats were administrated with 11β-HSD2 inhibitor CBX (2.4mg/kg) or saline from GD7.5 to GD17.5. Urine was collected from GD18.5 to GD19.5. After determination of arterial BP, the rats were sacrificed on GD20.5 for collection of blood and tissues. **A**, MAP measured from GD 9.5 until GD 17.5. **B**, SBP measured on GD20.5. **C**, the circulatory sFlt1 and sEng levels in the rat model. **D**, protein/creatinine (mg/mg) in urine in the rat model . **E**, morphology of glomeruli stained by H&E and PAS. Left panel: the representative images (400×). Right panel: histopathological score of glomerular pathology. **F**, doppler ultrasonography. Upper panel: the representative images of SA in implantation sites, canal in placentas and fetal UmbA visualized by ultrasound biomicroscopy. Lower panel: cumulative data of the PSV of SA, Canal and UmbA. $*p < 0.05$, $**p < 0.01$, $***p < 0.001$, $****p < 0.0001$. Con: control.

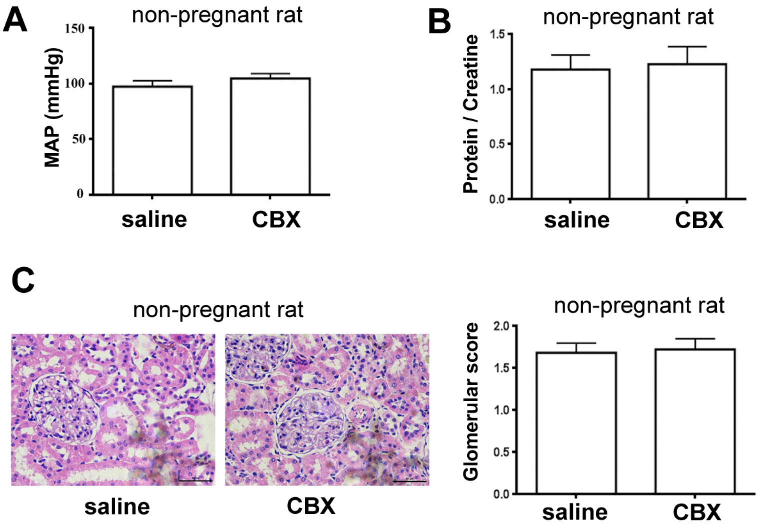

Figure S2. The effects of CBX on blood pressure and renal morphology in nonpregnant rats. Nonpregnant female rats were randomly divided into two groups saline or CBX (n=5 in each group). The rats were injected saline or CBX at 2.4mg/kg once a day for 10 days. Rats of saline group received same volume of saline. **A**, MAP. **B**, protein/creatinine (mg/mg) in urine. **C**, morphology of glomeruli stained by H&E. Left panel: the representative images (400×). Right panel: histopathological score of glomerular pathology.

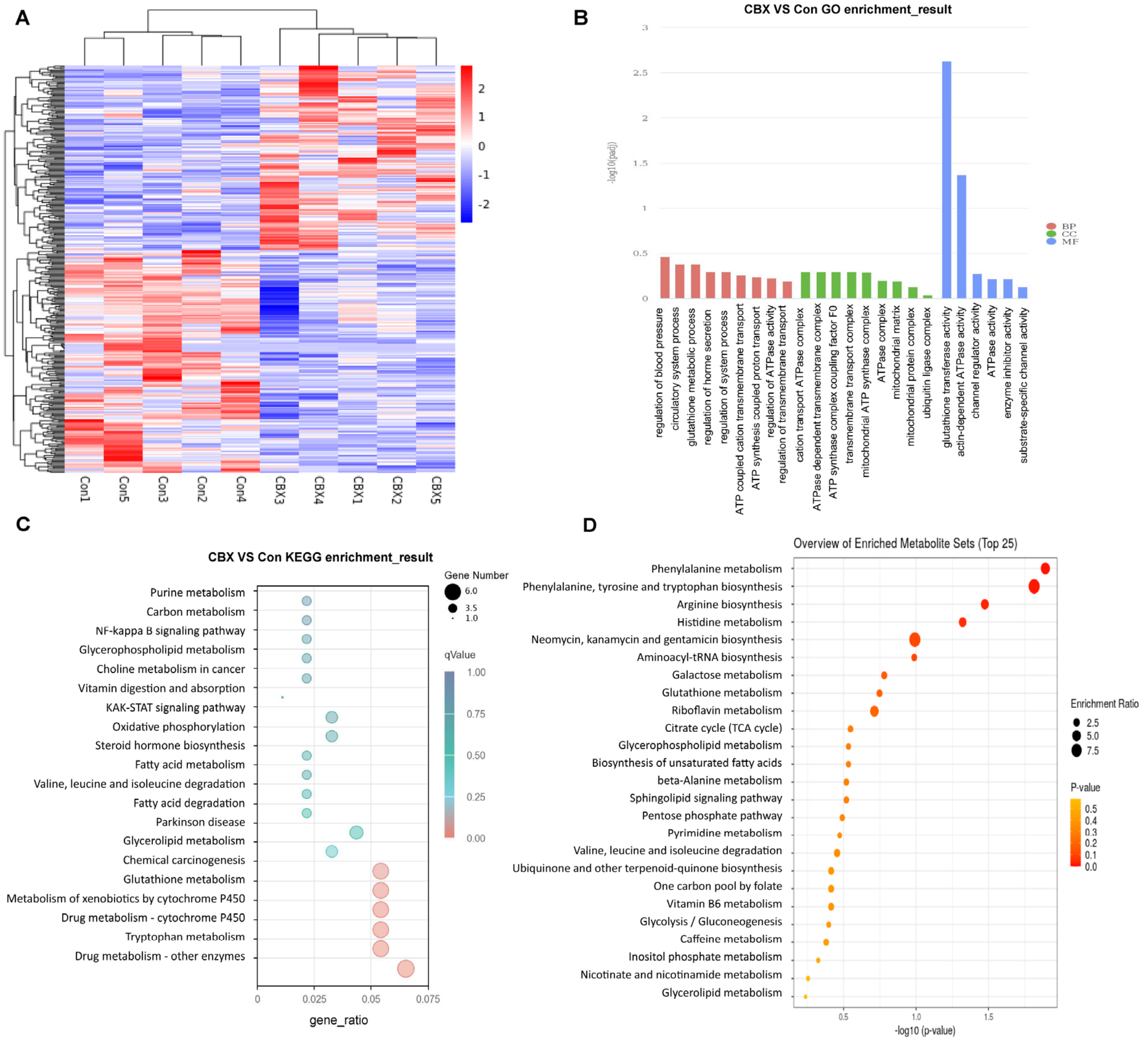

Figure S3. Heatmap and pathway enrichment of transcriptomics and metabolomics of the placentas in rat PE-like model. Pregnant rats were administrated with CBX (2.4mg/kg) or saline from GD7.5 to GD17.5. The placentas were collected on GD20.5 for RNA-seq and untargeted metabolomics. **A**, the differential genes analyzed by RNA-seq. Left panel: heat map of the differential genes (CBX VS Con: P value<0.05, Fold Change>1.5). Right panel: statistical map of up and down regulated differential genes. **B**, GO enrichment analysis in transcriptomics. **C**, KEGG enrichment analysis in transcriptomics. **D**, KEGG enrichment analysis in metabolomics.

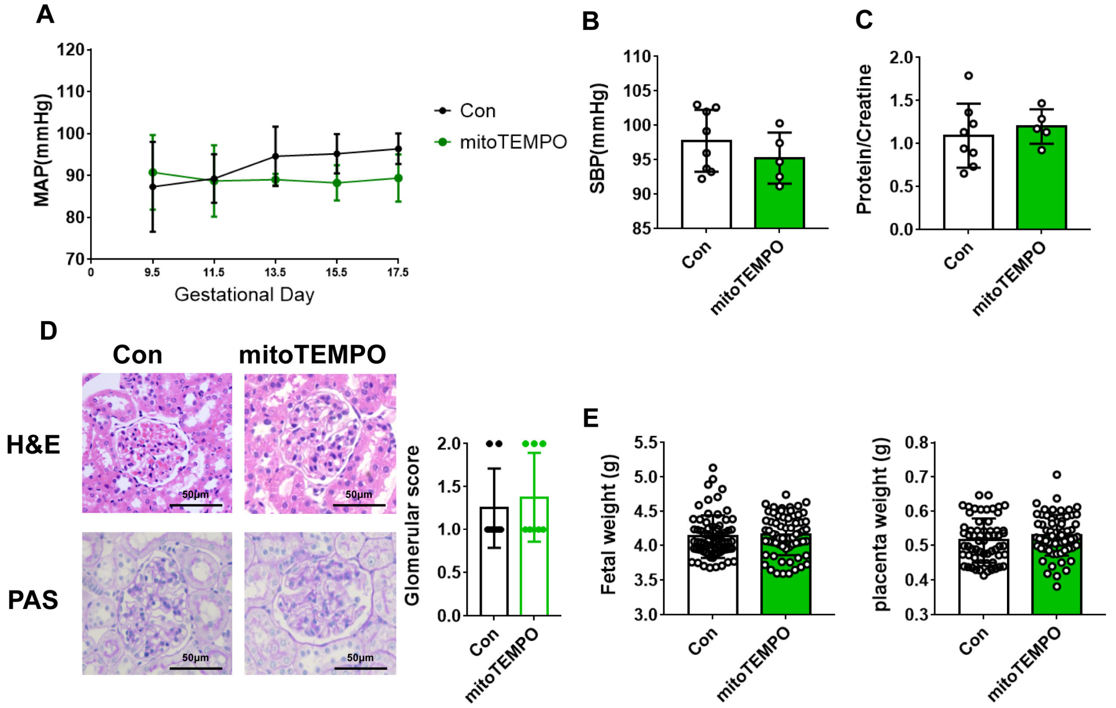

Figure S4. The effects of MitoTEMPO alone on blood pressure, renal morphology, and fetal and placental weight in pregnant rats. Pregnant rats were administrated with mitoTEMPO (1mg/kg) from GD7.5 to GD17.5. Urine was collected from GD18.5 to GD19.5. After determination of arterial BP, the rats were sacrificed on GD20.5 for collection of blood and tissues. **A**, MAP measured from GD 9.5 until GD 17.5. **B**, SBP measured on GD20.5. **C**, protein/creatinine (mg/mg) in urine in the rat model. **D**, morphology of glomeruli stained by H&E and PAS. Left panel: the representative images (400×). Right panel: histopathological score of glomerular pathology. **E**, fetal and placental weight from 8 dams (each group) measured on GD 20.5. *$p < 0.05$, **$p < 0.01$, ***$p < 0.001$, ****$p < 0.0001$. Con: control.

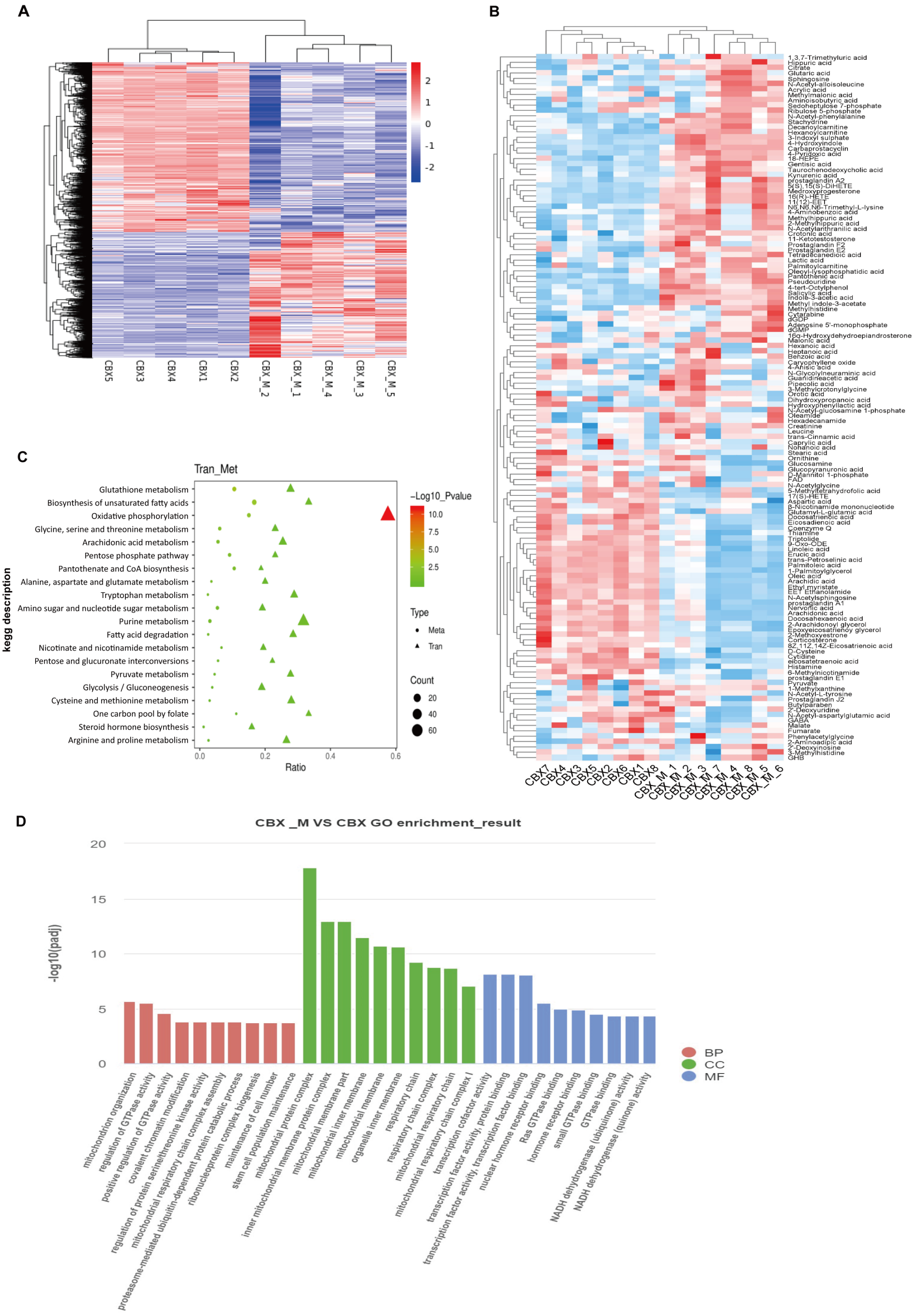

Figure S5. Transcriptomics and metabolomics of the placentas in the PE-like model with mitoTEMPO treatment. Pregnant rats were administrated with CBX (2.4mg/kg) and CBX combined with mitoTEMPO (1mg/kg) from GD7.5 to GD17.5.

The placentas were collected on GD20.5 for RNA-seq and untargeted metabolomics. **A**, Cluster heat map of the differential genes (CBX_M VS CBX: P value<0.05, Fold Change>1.5). Rows are CBX and CBX_M arms and columns are differential genes, respectively. **B**, Cluster heat map of the differential metabolites (CBX_M VS CBX: VIP>1, P value<0.05, Fold Change>1.5). Rows are CBX and CBX_M arms and columns are differential metabolites, respectively. **C**, KEGG enrichment analysis combined transcriptomics with metabolomics. **D**, GO enrichment analysis in transcriptomics. CBX_M: CBX combined with mitoTEMPO treatment; Tran: transcriptomics; Met: metabolomics

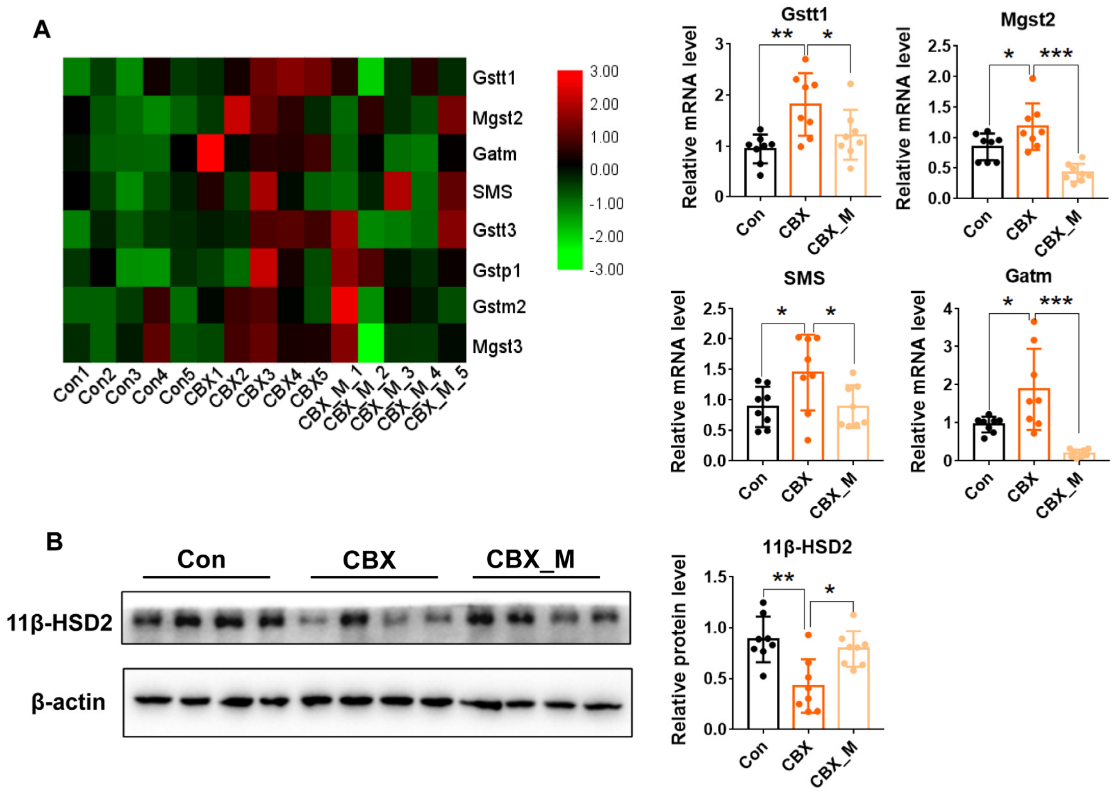

Figure S6. The expression level of the genes in glutathione metabolism pathway and 11β-HSD2 expression in the PE-like model with mitoTEMPO treatment.

Pregnant rats were administrated with CBX (2.4mg/kg), CBX combined with mitoTEMPO(1mg/kg) or saline from GD7.5 to GD17.5. The rats were sacrificed on GD20.5 for collection of blood and placental tissues. **A**, the transcriptional levels of the genes that related to glutathione metabolism. Left panel: heatmap of the genes related to glutathione metabolism in RNA-seq. Right panel: cumulative data of Q-PCR analysis. **B**, 11β-HSD2 protein expression level. Left panel: representative images of western blotting. Right panel: cumulative data of each protein expression level. $^{*}p < 0.05$, $^{**}p < 0.01$, $^{***}p < 0.001$, $^{****}p < 0.0001$. Con: control; CBX_M: CBX combined with mitoTEMPO treatment.
